# MR1-Restricted T Cells in Cancer Immunotherapy

**DOI:** 10.3390/cancers12082145

**Published:** 2020-08-03

**Authors:** Pedro Flores-Villanueva, Navid Sobhani, Xu Wang, Yong Li

**Affiliations:** Department of Medicine, Section of Epidemiology and Population Sciences, Baylor College of Medicine, Houston, TX 77030, USA; Navid.Sobhani@bcm.edu (N.S.); Xu.Wang@bcm.edu (X.W.)

**Keywords:** MR1, T-cell receptor, immunotherapy, riboflavin, folic acid, glycolysis, neoantigen

## Abstract

Major histocompatibility complex class I-related (MR1) was first identified as a cell membrane protein involved in the development and expansion of a unique set of T cells expressing an invariant T-cell receptor (TCR) α-chain. These cells were initially discovered in mucosal tissues, such as the intestinal mucosa, so they are called mucosal-associated invariant T (MAIT) cells. MR1 senses the presence of intermediate metabolites of riboflavin and folic acid synthesis that have been chemically modified by the side-products of glycolysis, glyoxal or methylglyoxal. These modified metabolites form complexes with MR1 and translocate from the endoplasmic reticulum to the plasma membrane where MAIT cells’ TCRs recognize them. Recent publications report that atypical MR1-restricted cytotoxic T cells, differing from MAIT cells in TCR usage, antigen, and transcription factor profile, recognize an as yet unknown cancer-specific metabolite presented by MR1 in cancer cells. This metabolite may represent another class of neoantigens, beyond the neo-peptides arising from altered tumor proteins. In an MR1-dependent manner, these MR1-restricted T cells, while sparing noncancerous cells, kill many cancer cell lines and attenuate cell-line-derived and patient-derived xenograft tumors. As MR1 is monomorphic and expressed in a wide range of cancer tissues, these findings raise the possibility of universal pan-cancer immunotherapies that are dependent on cancer metabolites.

## 1. Introduction

Recently, there has been increasing interest in the role of the microbiome in developing and activating the mucosal host immune response against tumor formation, infections, and inflammation [1,2,3,4,5,6]. The major histocompatibility complex (MHC) class I-related (MR1) is a cell membrane protein expressed in cells infected by certain types of bacteria or in cells under metabolic stress [7,8,9,10,11]. Tumor cells also express MR1 [12,13]. Unlike the highly polymorphic MHC-I molecules, MR1 is monomorphic. Rather than MHC-I binding 8–10-amino acid peptides, MR1 binds small metabolite molecules derived from bacterial biosynthesis of riboflavin (vitamin B2) or folic acid (vitamin B9) [7,8,14]. The binding of those vitamin B metabolites to MR1 requires their modification through chemical reactions with glyoxal or methylglyoxal, which are generated endogenously by glycolysis or derived from exogenous food sources [7,15,16,17,18,19,20]. Notably, bacteria and tumor cells use glycolysis as a source of ATP [21,22]. The MR1-metabolite complex is then presented to a class of T cells, called mucosal-associated invariant T (MAIT) cells [23], as they were originally discovered to be preferentially located in the gut lamina propria of humans and mice [7,8]. Significant advances have been made in the understanding of MAIT cell functions following the discovery of MR1 ligands [7,14]. Recent publications report that a subpopulation of MR1-restricted cytotoxic T cells recognizes an as yet unknown cancer-specific metabolite presented by MR1 in cancer cells [24]. These atypical MR1-restricted T cells (called MR1T), with their distinctive TCRs, represent a unique opportunity for the development of pan-cancer therapeutic agents. In this work, we review the subsets of MR1-restricted T cells, their antigen presentation and cancer biology, and their potential applications in cancer immunotherapy.

## 2. The Biology of MR1 in MAIT Cells

The gene encoding MR1 is located in chromosome region 1q25.3 and exists in three isoforms; however, the protein is monomorphic [25]. MR1 can form complexes with β2-microglobulin (β2M), an 11.8-kD protein interacting and stabilizing MR1, similar to classical MHC-I. It is clear that the association with β2M is essential for MR1 plasma membrane expression and for its ability to bind antigens [7,8]. That is one of the reasons why MR1 is regarded as an MHC I-related molecule [7,8]. The exception to the rule is the isoform 3/MR1B, which does not associate with β2M [25]. The mature MR1 monomer is located in intracellular membranes and senses metabolic changes [7,8,14,18,19,20,21,22]. As vitamin B metabolites are captured through their receptors, expressed by mammalian cells, they react with glyoxal or methylglyoxal, which are produced by cells undergoing metabolic stress, including intense glycolysis. The appropriate metabolite binds to MR1 along with β2M, then this MR1-β2M-antigen complex is translocated to the plasma membrane [7,8]. Currently known metabolites that are capable of binding MR1 are byproducts of the spontaneous chemical reaction between metabolites resulting from the microbial synthesis of folic acid (vitamin B9) or riboflavin (vitamin B2) and the chemical reaction of those metabolites with byproducts of glycolysis—either methylglyoxal or glyoxal [7,8,14,15,16,17]. The results of this chemical reaction are the MR1-binding antigen metabolites: 5-OP-RU (5-(2-oxopropylideneamino)-6-D-ribitylaminouracil) or 5-OE-RU [5-(2-oxoethylideneamino)-6-D-ribitylaminouracil], products of vitamin B2 biosynthesis, and 6-formylpterin (6-FP), a product of the folic acid biosynthesis [7,14]. 5-OP-RU influences the thymic development of MAIT cells in mice [26]. Interestingly, those three metabolites can fold MR1 tetramers, but only 5-OP-RU and 5-OE-RU, in complex with MR1, are able to activate MAIT cells [7,14]. Notably, these metabolite-producing pathways, vitamin B9 and B2 synthesis and the production of glyoxal and methylglyoxal, exist in several microbes and in glycolysis; the latter is a characteristic metabolic condition in tumor cells under aerobic and anaerobic conditions (the Warburg effect) [22,25,27]. It is intriguing that MR1-tetramers refolded with 5-OP-RU or 5-OE-RU can efficiently bind all human MAIT cells circulating in the blood [7,24]. Adding to this conundrum, the list of MR1 targets has recently been expanded to include 3-formylsalicylic acid, diclofenac, and methotrexate intermediate metabolites [14,28,29]. 

MAIT cells are activated by MR1 in complex with metabolites produced by bacteria and fungi of the human microbiome, especially the gut microbiota [27,28,29,30]. In addition, they are capable of mounting an immune response against tumor antigens [12,13,24,28,30]. It appears that the renewal of the MAIT cell compartment is maintained by constant stimulation of MAIT cells or their precursors in the mucosa. MAIT cell activation requires TCR recognition of the metabolite bound to the MR1 molecule for its activation [7,8]. It appears that the metabolites cannot activate MAIT cells without MR1, yet MAIT cells can be activated by different cytokines (IL-12 and IL-18) through an MR1-independent mechanism [7,8,9,10].

## 3. Classical MAIT Cells with TRAV1-2 

**Figure 1:**
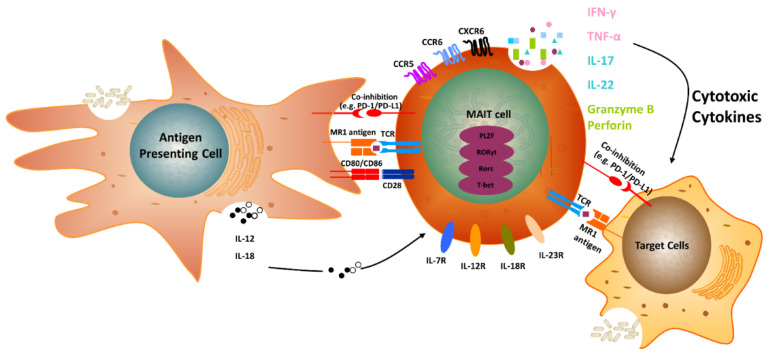
Mechanisms of MAIT cell activation. The ligands derived from microbes are presented by MR1 to activate MAIT cells. Activated MAIT cells secrete Th1/Th17 cytokines, such as IFN-γ, TNF-α, IL-17, and IL-22, and cytolytic enzymes, such as granzyme B, and perforin, to kill infected cells and recruit other immune cells. Co-inhibitory molecules, such as PD-L1, temper the MAIT cell response.

**Figure 2:**
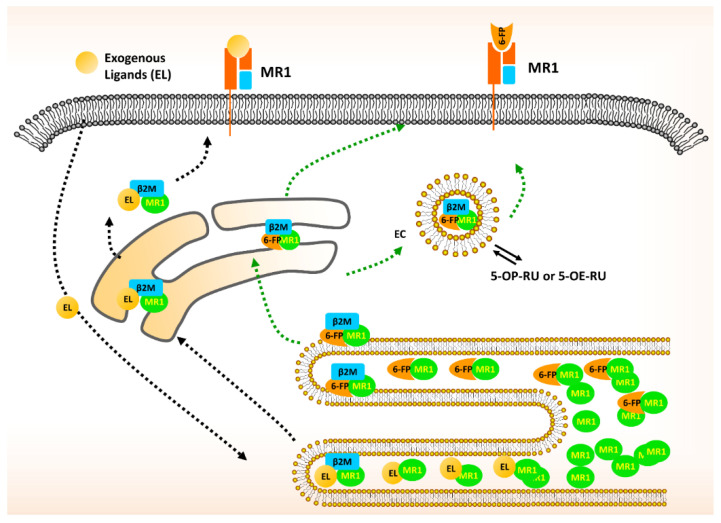
Schematic diagram of MR1 trafficking and presentation. MR1 is synthesized and accumulates in the endoplasmic reticulum (ER) in an unfolded state. In the presence of ligands derived from extracellular bacteria or tumor cells, MR1 binds to exogenous ligands and presents them on to the plasma membrane through the ER and Golgi (black dashed arrow). However, vitamin B metabolites, such as 6-FP, derived from an intracellular infection, utilize an alternative pathway to present the MR1-ligand complex at the cell surface. The MR1-ligand binding complex either passes to the plasma membrane or translocates into endosomes. In a low pH environment, the intracellular ligands are exchanged with exogenous ligands, such as 5-OP-RU or 5-OE-RU, and the MR1-ligand complex then translocates to the plasma membrane to be recognized by the TCR (green dashed arrow). β2M, β2-microglobulin; 6-FP, 6-formylpterin.

MAIT cells are T cells expressing an invariant T-cell receptor (TCR) α-chain [9]. Immunologists are currently debating whether all MAIT cells carry an invariant TCR α-chain. This invariant TCR α-chain is usually comprised of a T-cell receptor-α variable (TRAV) 1-2 region and a joining region in their T-cell receptor-α (TRAJ) that can be, in order of frequency: TRAJ33, TRAJ12, and TRAJ20 [30,31,32,33,34] (Figure 1). Interestingly, the sequences of all of these TRAJ segments contain a tyrosine at position 95, which is involved in metabolite-antigen recognition by TRAJ33 [31,35]. MAIT cells are mostly located in the liver, gut lamina propria, lung, spleen, lymph nodes, thymus, kidney, and skin [28]. About 1% to 10% of T cells in the blood are MAIT cells [28]. TRAV1-2^+^ MAIT-cells can be CD4^-^ CD8^-^, CD4^+^ CD8^-^, CD4^-^ CD8αα^+^, or CD4^-^ CD8αβ^+^ [28]. The most frequent TRAV1-2^+^ MAIT-cells are CD8αα^+^ [35]. Given that the CD8 molecules seem to be involved in the recognition of MR1, it will be interesting to know how CD8^-^ MAIT cells stabilize that interaction. All TRAV1-2^+^ MAIT cells express both CD161 and CD26 at high levels and the T-cell memory marker CD45RO [28,35]. It is unknown whether they express immune checkpoint inhibitory surface molecules (e.g., PD1, CTLA4, TIM3, LAG3, TIGIT, VISTA, or B7/H3), co-stimulatory molecules (e.g., CD80, CD86, OX40, ICOS, GITR, 4-1BB, or CD40), or other molecules found in the tumor microenvironment, such as IDO or TLR. Classical MAIT cells express the transcription factors, promyelocytic leukemia zinc finger (PLZF) and RAR-related orphan receptor γt (RORγt) [28]. The latter transcription factor is also expressed by regulatory T cells and interleukin-17 (IL-17)-producing helper T cells (Th17) [36]. 5-OP-RU is a major antigen recognized by MR1 and the MAIT TCRs. MR1 also binds 6-FP, a byproduct of folic acid biosynthesis [7,14] (Figure 2). The cytotoxic capacity of MAIT cells is likely due to their ability to express perforin and granzyme B upon activation [28]. MAIT cells also express granulysin [28]. It is unknown whether they also use the FAS/FAS ligand (FASL) or other pathways for their cytotoxic function. MAIT cells can produce, upon TCR-activation, IL-17, along with IL-22, TNF, IL-2, and interferon-γ (IFN-γ). IL-17 may increase the proliferative capacity of different types of tumors [28].

## 4. Non-classical MAIT Cells without TRAV1-2 

**Figure 3:**
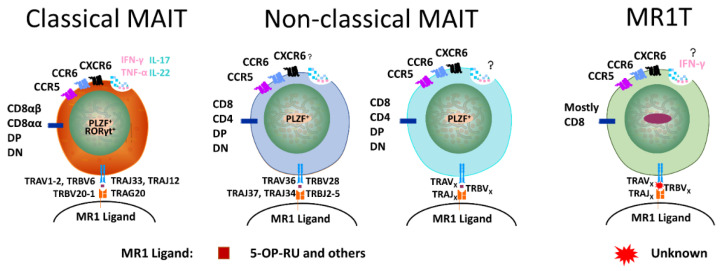
Main characteristics of subsets of human MR1-restricted T cells with αβ TCRs. Classical MAIT cells express TCRs with TRAV1-2 and PLZF. Non-classical MAIT cells express PLZF but not TCRs with TRAV1-2. Atypical MR-1 restricted T (MR1T) cells express neither.

There are MAIT cells that have no TRAV1-2 in their TCRs (TRAV1-2^-^) [28,37]. These non-classical MAIT cells account for less than 0.01% of circulating T cells, yet they share many features with TRAV1-2^+^ classical MAIT cells [28]. Their functions are less well known and some of them have shown reactivity against several tumors expressing MR1. We summarize the characteristic features of non-classical MAIT cells in Table 1. Similar to classical MAIT cells, non-classical MAIT cells express PLZF, CD161, and IL-18R, as well as recognize 5-OP-RU as an antigen; however, the TCRs expressed by non-classical MAIT cells are significantly different from those expressed by classical MAIT cells, with one subgroup showing more diverse TCRs than the other (Table 1; Figure 3).

## 5. MAIT Cells and Cancer

Clinical studies of the role of MAIT cells in solid tumors are mostly focused on hepatocellular carcinoma (HCC) and colorectal cancer (CRC). It is remarkable that MAIT cells are abundant in normal hepatic tissues (~50% of T cells in the liver are MAIT cells; Table 1) but are decreased in HCC tumor tissues [12,38,39]. Duan et al. reported that tumor-educated MAIT cells in HCC expressed higher levels of PD1, CTLA4, and TIM3, yet produced lower levels of IFNγ, IL-17, granzyme B, and perforin [38]. Thus, HCC-infiltrating MAIT cells were functionally compromised and likely reprogrammed from being tumor-suppressive to being tumor-promoting [38]. In contrast, more MAIT cells infiltrate CRC tumor tissues than infiltrate healthy colorectal tissues [40,41,42,43]. MAIT cells in CRC express higher levels of IL-17A and lower levels of IFN-γ than those in healthy tissue [42]. Perhaps MAIT cells in CRC patients differentiate towards a Th17 phenotype. A transwell assay showed an increase in the migration of MAIT cells towards mucosal cancer cells [44]. In addition, it is known that normal cells express low levels of MR1 [7,8,24]. Perhaps the inflammatory milieu of the tumor is enriched in factors that increase MR1 expression in tumor tissues, including both tumor cells and stromal cells. Thus, MAIT’s MR1-specific cytotoxic activity may reach many cell types within a tumor, disrupting the integrity of the tumor stroma, vessels, and lymphoid tissues, and facilitating the dissemination of tumor cells beyond the tumor milieu. 

The frequency of circulating MAIT cells in patients with non-mucosa-associated solid tumors (e.g., kidney, breast, and thyroid cancers) was found to be high [44]. Interestingly, in kidney, urothelial, and prostate cancers, the high MR1 mRNA expression level is associated with better probability of survival than those with low levels of MR1 mRNA expression [28]. In patients suffering from breast, thyroid, and lung cancers, MR1 expression level is not associated with survival [28]. Worth mentioning is the fact that, in several different cancers, including glioma, melanoma, colorectal, stomach, liver, pancreatic, head and neck, cervical, endometrial, and ovarian cancers, a high MR1 expression level is associated with decreased probability of survival [28]. It should be noted that levels of MR1 expression might not correlate with levels of MAIT cell infiltration into tumors; these two parameters should be investigated in parallel to provide clear conclusions. More studies using larger cohorts and complete histopathological classification of the tumors will provide a precise understanding of the role of MAIT cells in the various cancers and the relationship to clinical outcomes.

In addition to solid tumors, it has been shown that multiple myeloma (MM) cell lines express MR1 in complex with vitamin B metabolites. Consequently, MAIT cells derived from healthy controls were able to efficiently kill MM cells in vitro [44]. Interestingly, in a study on newly diagnosed MM patients, the frequency of MAIT cells was lower than that in healthy controls [44]. Moreover, the percentages of CD8^+^ and CD8^-^ CD4^-^ MAIT cells in MM patients were lower than in healthy controls [44]. Thus, MAIT cells may play a protective role in MM. In addition, a study using peripheral blood from 91 patients suffering from myelogenous leukemia and 20 healthy controls showed that MAIT cells displayed TCR-activated cytotoxic activity [44]. Indeed, after in vitro activation they were cytotoxic to myelogenous leukemia cells [44].

## 6. MAIT Cells and IL-17 Family Members

It is interesting that MAIT cells or different subsets of MAIT cells appear to play dichotomous roles, promoting the growth of or eliminating cancer cells [12,13,28]. One school of thought is that MAIT cells may promote cancer growth at early stages of tumorigenesis and eliminate cancer cells at late stages [12,13,28]. We reasoned that this dual role might have to do with the subtype of MAIT cells infiltrating the tumor and/or the tumor microenvironment. 

Such a dichotomous role of MAIT cells may have something to do with their capacity to secrete IL-17 and/or the expression levels and isoforms of IL-17 receptors (IL-17RA to IL-17RE) within tumors [45,46]. IL-17 family members are widely reported to have dual roles in tumorigenesis [45,46]. The IL-17 family is comprised of six molecules (IL-17A to IL-17F, including IL-17E, which is also called IL-25) [45,46]. They display high homology, are secreted as homodimers or heterodimers, and have different binding affinities for IL-17Rs [45,46]. For instance, IL-17A may exhibit 20-times higher-affinity binding than IL-17F to, presumably, the IL-17RA/RC heterodimer, providing a signal that is more robust than that of IL-17F [45,46]. 

That MAIT cells secrete IL-17 that may explain that its tumor-growth-promoting ability is its role in promoting the tumor microenvironment. IL-17 induces the secretion of CCL2, CXCL1, CXCL5, CXCL6, and CXCL8 from several types of cells that infiltrate the tumor, and subsequently, it recruits myeloid-derived suppressor cells, including tumor-associated macrophages [45,46]. On the other hand, IL-17 family members may elicit immune defense against tumors by orchestrating anti-tumor activity by promoting the secretion of chemokines that attract tumor-suppressive lymphocytes, such as CD8^+^ T cells, to the tumor [45,46]. In addition to IL-17 production for host defense against tumors, MAIT cells are able to directly eliminate tumor cells through the expression of perforin and granzyme B after activation through their TCRs, which are specific against target tumor cells expressing MR1 in complex with metabolite antigens in the plasma membrane [7,8,9,10,11,12,13,14,28,30,31,41,47]. Moreover, IL-17A is known to stimulate tumor cell proliferation and metastasis in non-small-cell lung cancer and breast cancer [48,49,50,51,52]. Perhaps, this is one of the mechanisms associated with the poor prognosis of HCC and CRC patients with tumors of IL-17A-expressing MAIT cells.

Until we know in detail how IL-17 family members, secreted from MAIT cells, act as factors to promote tumor cell growth or to enhance tumor cell killing and MAIT activation, we cannot propose a safe intervention to antagonize or stimulate IL-17 production, secretion, and functions for cancer therapies. Given the fact that vitamins B2 and B9 are important for all mammalian cell metabolic functions, targeting the inhibition or activation of their receptors is unlikely to be beneficial for the inhibition or activation of MAIT cells [53,54].

## 7. Atypical MR1-Restricted T cells 

In 2017, Lepore et al. reported a novel population of MR1-restricted T cells that expressed diverse TCR-α and TCR-β genes, yet they were unable to recognize previously identified microbial or folate-derived ligands of MR1 [55]. These cells were named MR1T cells. The frequency of circulating MR1T cells is estimated to be 0.02% of total T cells [55]. Six MR1T lines were cloned and their TCR sequenced (Table 2). All MR1T cells expressed IFN-γ, and some expressed other cytokines, as in Th1 cells (IL-2, TNF-α, and TNF-β), Th2 cells (IL-3, IL-4, IL-5, IL-6, IL-10, IL-13), and Th17 cells (IL-17A, G-CSF, GM-CSF), supporting remarkable functional plasticity [55]. Two MR1T cell clones expressed transcription factors FOXP3, FOSL2, and IRF4 [55]. Three years later, Crowther et al. reported a single clone of MR1T cells that mediated the in vitro killing of cancer cells and the in vivo regression of autologous and non-autologous tumors [24]. 

Crowther et al. used A549 lung tumor cells co-cultured with a patient’s peripheral blood mononuclear cells and isolated a T cell clone (MC.7.G5; Table 2) that was able to kill A549 cells and sequenced its TCR. This MR1T clone expressed IFN-γ and TNF-α upon activation; no information was provided regarding PLZF [24], similar to the Leport et al. report [55]. Crowther et al. made cDNA constructs with the TCR for the transfection and expression of those TCRs in cytotoxic T cells and performed comprehensive genome-wide knockout screening using the CRISPR-Cas9 system. They were able to find six genes (MR1, β2M, RFX, RFXANK, RFXAP, and STAT6) in HEK293T cells essential for the activation of MC.7.G5 cells [24]. They discovered that MR1 is one of the essential proteins needed for MR1T TCR-mediated targeting of cancer cells while sparing noncancerous cells [24]. Clone MC.7.G5 expresses TCRs capable of recognizing MR1-metabolite complexes in the plasma membrane of several different types of tumor cell lines and primary tumor cells in vitro [24]. However, as mentioned above, these metabolites (i.e., antigens recognized by the TCR) are currently unknown [24].

Crower et al. showed that MR1T cells induced cell death in autologous and non-autologous melanoma, as well as lung, breast, colon, prostate, ovarian, and hematological cancer cell lines [24]. The cytotoxicity of MR1T cells depends on: (1) the expression of MR1 in complex with β2M on the tumor-cell surface; (2) the unknown antigen or antigens that are restricted by MR1-β2M; (3) the TCR of the MR1T cells that recognizes the MR1-β2M-ligand complex. We cannot completely rule out that the MR1T TCRs require an additional and unidentified membrane molecule for stable interaction with MR1–β2M–ligand. The chemical natures of the antigen or antigens within tumor cells bound to the MR1 of tumor cells and the TCR of MR1T cells remain elusive, but it is possible that they are unstable intermediates resulting from the reaction of vitamin B2 (riboflavin) intermediates with glyoxal or methylglyoxal (see below). 

## 8. TCR Antigens of MAIT Versus MR1T Cells

It is important to mention that neither riboflavin (vitamin B2) nor folic acid (vitamin B9) is produced by mammalian cells [25,27,56]. Thus, microbial infection or colonization with microorganisms expressing functional vitamin B-metabolizing enzymes might be required to produced 5-OP-RU and 5-OE-RU. Circulating unstable metabolites of vitamin B2 and B9 produced in microbial-colonized mucosa may reach the tumor cell milieu and be captured by specific receptors and translocated to the intracellular space, where they react with glyoxal or methylglyoxal to generate the MR1-binding tumor metabolites [8,57]. Whether the resulting MR1-metabolite complex is able to activate MAIT cells in vivo is not clear because 5-OP-RU and 5-OE-RU, but not 6-FP, can activate MAIT cells in vitro [7,14]. 

In regard to the manner by which MAIT cells’ TCR α- and β-chains dock the MR1-antigen metabolite complex, Patel et al. [14] described the astonishing similarity of this interaction with that of conventional TCR α- and β-chains with MHC class I/peptide complexes [14]. However, upon interaction of MAIT cells’ TCR with MR1-antigen complexes, the former undergoes remodeling of the antigen-binding cleft, a feature observed in innate pattern-recognition receptors [14]. Thus, the TCR of MAIT cells might be an innate-like pattern-recognition receptor targeted towards vitamin B metabolites, and MAIT cells are considered reminiscent of innate immunity, bridging innate and cognate mechanisms of ligand recognition. In support of the innate-like nature of MAIT cells’ TCRs is the fact that they recognize the monomorphic MR1 in complex with a limited number of metabolite antigens [7,14]. In contrast, the classical MHC-I proteins are highly polymorphic and the peptides they bind are diverse, depending on the flexibility of the TCR V region, which is often sufficient to allow the TCR to interact with numerous MHC-I/peptide complexes [58].

We have limited knowledge of the tumor antigens recognized by the αβ TCRs of MR1T. The anti-cancer MR1T clones isolated by Crowther et al. did not recognize MR1 tetramer complexes with the microbial-derived T cell activator 5-OP-RU [24,35]. Yet, the recognition of target cancer cells by MR1T was reduced when loaded with either MAIT-activating bacteria, *Mycobacterium smegmatis* or *Salmonella enterica*, or the MR1 ligand acetyl-6-FP [24]. These results indicate that the as yet unknown ligand or ligands restricted by MR1, and thereby recognized by MR1T cells, have a similar structure to acetyl-6-FP and other MAIT TCR ligands. In addition to being byproducts of glycolysis, glyoxal and methylglyoxal come from food sources, yet it is tantalizing to speculate that MR1-expressing cancer cells undergo increased glycolysis to produce glyoxal and methylglyoxal, which react with vitamin B metabolites to form the antigen or antigens that bind MR1 and are recognized by the TCRs of MR1T cells. As the unknown metabolite antigen or antigens presented by MR1 are specific to or associated with cancer, they may represent a novel class of neoantigens, beyond the neo-peptides arising from altered tumor proteins and presented by classical MHC-I or MHC-II. 

## 9. MR1-Restricted γδ T Cells

Virtually all MR1-restricted T cells were reported to have αβ TCRs until late 2019, when Le Nours et al. reported a class of γδ TCRs in MR1-restricted T cells [59] (Table 1). This group detected MR1-tetramer^+^ γδ T cells that accounted for <0.001% to 0.1% of CD3^+^ circulating T cells and <0.1% to 5% of γδ T cells. MR1-5-OP-RU tetramer^+^ γδ T cells were mostly CD4^−^CD8α^−^ or CD8α^+^ with variable CD161 expression, resembling other cells of the γδ T cell lineage [59]. MR1-restricted γδ T cells were detected by staining in the liver, stomach, lung, and duodenum of healthy subjects and were enriched in a celiac duodenum and a Merkel cell carcinoma [59]. The group solved the crystal structure of a γδ TCR–MR1–5-OP-RU complex and found that the γδ TCR binds underneath the MR1 antigen-binding cleft, rather than binding to the presented antigen within the cleft [59]. Thus, MR1-restricted γδ TCRs in T cells can adopt diverse binding modes with MR1, representing noteworthy progress for both γδ T cell and MR1 biology. We classify these γδ T cells as an independent subset of MR1-restricted T cells (Table 1).

## 10. MR1T Cells in Cancer Immunotherapy

**Figure 4:**
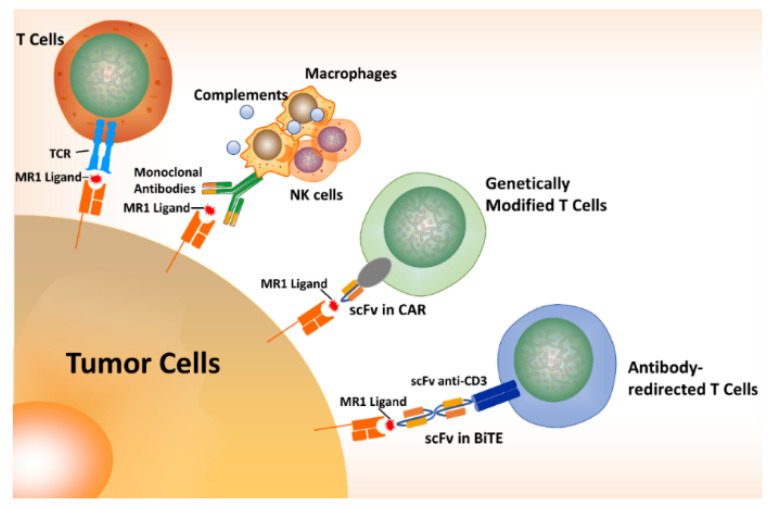
MR1-dependent strategies for cancer treatment. There are at least four routes to targeting MR1-expressing tumor cells. (1) MR1-mediated tumor cell lysis is mediated by the recognition of MR1-ligand by a TCR. (2) An antibody targeting MR1-ligand induces phagocytosis and complements activation and antibody-dependent cellular cytotoxicity (ADCC). (3) The scFv of above antibody is engineered into CAR T cells. (4) BiTE redirected-T cells have two scFvs to recognize both CD3^+^ T cells and MR1-ligand.

The discovery of MR1T opens the door to translating the findings of MR1-restricted T cells to clinical application. The potential of MAIT cells in immunotherapy is limited, as they are essential for host immunity to bacterial infections, and their antigens are not specific to or associated with cancer [28]. The nature of the as yet unknown neoantigen to MR1T prompts us to envision four potential routes of MR1T cells in cancer immunotherapy: TCR gene therapy, monoclonal antibody therapy, chimeric antigen receptor (CAR) T therapy, and bispecific T cell engager (BiTE) therapy (Figure 4). First, autologous T cells are genetically engineered with the TCRα and TCRβ subunits of MR1T cells (such as those listed in Table 2). Second, a monoclonal antibody is developed to bind both MR1 and its tumor-specific antigens to induce antibody-dependent cytotoxicity. Third, the single-chain variable fragment (scFv) of the above antibody is used in CAR T cells to target the MR1-antigen complex from tumor cells. Finally, the above scFv is fused to the scFv of an antibody against CD3 in a BiTE design to attract CD3^+^ T cells. The last three approaches would require us to identify the tumor-specific antigen or antigens first before generating the monoclonal antibodies. All these potential therapeutic approaches are dependent on MR1 expressed on the tumor cell surface and bound to the cancer-specific metabolites (i.e., neoantigens) but independent of antigen-presenting cell processing. A less appealing use of MR1T cells is to increase the generation or accumulation of the as yet unknown antigen in tumor cells as vaccines or treatments. This mechanism of action in immunotherapy has been attempted using neoantigens based on mutant tumor-protein-derived peptides. Yet, given the small size and the potentially transient nature of these metabolite neoantigens for MR1T cells, this approach is suboptimal.

## 11. Concluding Remarks

Immune surveillance is the mechanism by which the immune system detects and destroys any threat to the body, such as microbial pathogens or cancer cells. MAIT cells are capable of activating or inactivating a great variety of immune cells and are believed to, in some circumstances, participate in immune surveillance [28]. The activation of classical and non-classical MAIT cells using ligand agonists from microbes, such as vitamin B metabolites, is of great interest in host defense. Most of our knowledge on MR1-restricted T cells in cancer comes from studies on MAIT cells, which have been shown to have a dichotomous role in prognosis [28]. However, MR1T cells, the atypical MR1-restricted T cells, have a predominant role in killing tumor cells while sparing noncancerous counterparts. As MR1 is monomorphic and expressed in a wide range of cancer tissues, a future MR1T cell–based immunological therapy against all MR1-expressing cancers can be hypothesized and pursued by academia and industry. A possible obstacle in translating MR1 findings is T cell exhaustion and anergy. If this is the case, a combination of an MR1T agent and an antibody blocking immune checkpoints, such as anti-PD1 or anti-CTLA4, might hold promise. Moreover, future clinical studies should embrace efficient biomarkers to select patients who may benefit the most from this type of treatment. 

## Figures and Tables

**Table 1:** Main Characteristics of Subsets of Human MR1-restricted T cells.

SUBSETS	Classical MAIT	Non-Classical MAIT	MR1T	γδ T
TRAV1-2	TRAV1-2^+^	TRAV1-2^-^		
Frequency in blood T cells	1%–10%	0.001%–0.01%	0.02%	<0.001%–0.1%
Presence in organs	Liver ~ 50%,gastrointestinal tract, lung, thymus, lymph nodes, spleen, kidney, skin	Unknown or absent		Liver, stomach, lung, and duodenum; disease tissues
Antigen	All 5-OP-RU; subset-specific recognition of other antigens		Unknown	5-OP-RU, Ac-6-FP
TCR diversity	TRAV1-2, TRAJ33 or 12 or 20, TRBV6 or 20-1	TRAV36, TRAJ34 or 37, TRBV28, TRBJ2-5	TRAV_X_, TRAJ_X_, TRBV_X_	TRGVx, TRDV1 (72%), TRDV3/5
Transcription factors	PLZF^+^ RORγt^+^	PLZF^+^	PLZF^-^	Diverse
Co-receptors^*^	CD8αβ, CD8αα, DN, CD4, DP	CD8, DN, CD4, or DP	CD8, CD161, DN	DN, CD8α with CD161
Cytokines secreted	IL-17A, TNF-α, IFN-γ, IL-2, IL-22	Unknown	IL-17A, TNF, IFN-γ, IL-2	IFN-γ, TNF-α, and others

* DP, double positive; DN, double negative.

**Table 2:** The CDR3 Sequences of TCRs in MR1T Cell Clones.

Clone	TCRα (Accession No)	TCRα Sequence VJ	TCRβ (Accession No)	TCRβ V(D)J Sequence
DGB129	TRAV29 (MF085365)	CAASLYNQGGKLIFGQGTELSVKP	TRBV12-4 (MF085366)	CASSYRGTEAFFGQGTRLTVV
DGB70	TRAV5 (MF085363)	CAETWTDRGSTLGRLYFGRGTQLTVWP	TRBV28 (MF085364)	CASSLGATGANEKLFFGSGTQLSVL
DGA28	TRAV25 (MF085361)	CAAAGGTSYGKLTFGQGTILTVHP	TRBV291 (MF085362)	CSVGAGQGPYTDTQYFGPGTRLTVL
JMA	TRAV27 (MF085369)	CAGENSGYALNFGKGTSLLVTP	TRBV25-1 (MF085370)	CASSQLYRDTSNTGELFFGEGSRLTVL
TC5A87	TRAV131 (MF085371)	CAANWSPQGNEKLTFGTGTRLTIIP	TRBV25-1 (MF085372)	CASSEYIQYSGNTIYFGEGSWLTVV
CH9A3	TRAV24 (MF085367)	CASGDSGYALNFGKGTSLLVTP	TRAV24 (MF085368)	CASSFDVGLPPLHFGNGTRLTVT
MC.7.G5	TRAV38.2/DV8TRAJ31 (MN782533)	CAYRSAVNARLMFGDGTQLVVKP	TRBV25.1TRBJ2.3 (MN782534)	CASSEARGLAEFTDTQYFGPGTRLTVL
